# The Role of Multimodality Imaging for Percutaneous Coronary Intervention in Patients With Chronic Total Occlusions

**DOI:** 10.3389/fcvm.2022.823091

**Published:** 2022-05-02

**Authors:** Eleonora Melotti, Marta Belmonte, Carlo Gigante, Vincenzo Mallia, Saima Mushtaq, Edoardo Conte, Danilo Neglia, Gianluca Pontone, Carlos Collet, Jeroen Sonck, Luca Grancini, Antonio L. Bartorelli, Daniele Andreini

**Affiliations:** ^1^Centro Cardiologico Monzino, Istituto di Ricerca e Cura a Carattere Scientifico (IRCCS), Milan, Italy; ^2^Fondazione Toscana G. Monasterio, Pisa, Italy; ^3^Istituto di Scienze della Vita Scuola Superiore Sant'Anna, Pisa, Italy; ^4^Cardiovascular Center Aalst, OLV-Clinic, Aalst, Belgium; ^5^Department of Advanced Biomedical Sciences, University of Naples Federico II, Naples, Italy; ^6^Department of Biomedical and Clinical Sciences “Luigi Sacco”, University of Milan, Milan, Italy

**Keywords:** multimodality imaging, chronic total occlusion (CTO), cardiac CT, cardiac magnetic resonance, single photon emission computed tomography (SPECT), echocardiography, percutaneous coronary intervention (PCI)

## Abstract

**Background:**

Percutaneous coronary intervention (PCI) of Chronic total occlusions (CTOs) has been traditionally considered a challenging procedure, with a lower success rate and a higher incidence of complications compared to non-CTO-PCI. An accurate and comprehensive evaluation of potential candidates for CTO-PCI is of great importance. Indeed, assessment of myocardial viability, left ventricular function, individual risk profile and coronary lesion complexity as well as detection of inducible ischemia are key information that should be integrated for a shared treatment decision and interventional strategy planning. In this regard, multimodality imaging can provide combined data that can be very useful for the decision-making algorithm and for planning percutaneous CTO recanalization.

**Aims:**

The purpose of this article is to appraise the value and limitations of several non-invasive imaging tools to provide relevant information about the anatomical characteristics and functional impact of CTOs that may be useful for the pre-procedural assessment and follow-up of candidates for CTO-PCI. They include echocardiography, coronary computed tomography angiography (CCTA), nuclear imaging, and cardiac magnetic resonance (CMR). As an example, CCTA can accurately delineate CTO location and length, distal coronary bed, vessel tortuosity and calcifications that can predict PCI success, whereas stress CMR, nuclear imaging and stress-CT can provide functional evaluation in terms of myocardial ischemia and viability and perfusion defect extension.

## Introduction

Chronic total occlusions (CTOs) are completely occluded coronary arteries with Thrombolysis In Myocardial Infarction (TIMI) 0 flow with an estimated duration of at least 3 months (1–3) and a prevalence in patients undergoing coronary angiography ranging between 15% and 25% (3–6). Although percutaneous coronary intervention of CTO (CTO-PCI) is often a challenging procedure, the success rate continues to improve thanks to significant advancements in technology, techniques and operator experience. However, its clinical benefits have been debated for several years and the decision whether to treat patients should be weighted against technical challenges, significant radiation exposure, contrast dose, associated costs, and an overall lower success rate when compared to non-CTO-PCI.

### Rationale of CTO Revascularization in Terms of Ischemia Relief, Symptom and Left Ventricular Function Improvement and Reduction of Major Adverse Cardiac Events

The aim of CTO-PCI is to improve myocardial perfusion of the corresponding territory and to relieve ischemia (7, 8). Indeed, viable myocardium subtended by a CTO is generally ischemic, regardless of the extent of collateralization (9, 10). Myocardial ischemia relief after successful CTO revascularization has several positive effects for patients that are discussed below.

With regard to angina and quality of life (QoL), only the DECISION CTO study failed to demonstrate superiority of PCI compared with pharmacological treatment (11). However, several limitations of the trial should be acknowledged including slow and early termination of enrollment, high percentage of cross-over in both arms, high frequency of PCI for non-CTO lesions and inclusion of patients with mild or absent symptoms (11). In contrast, the EuroCTO study (12) and the IMPACTOR CTO study (13) showed, at 1-year follow-up, advantages of percutaneous revascularization in reducing angina and improving QoL assessed with the Seattle Angina Questionnaire (SAQ). Further confirmation of the efficacy of percutaneous revascularization in terms of improvement of symptoms comes from a recent study conducted by Hirai et al. In 1,000 consecutive patients with high-grade refractory angina, they showed that successful CTO-PCI led to higher improvement in the SAQ Angina Frequency and SAQ Summary Scores compared with unsuccessful PCI (14). This suggests that patients who may receive greater benefit from CTO-PCI are those with the highest level of ischemia. Refractory angina in patients with CTO may cause psychological distress and a depressive state. In this regard, the OPEN-CTO registry demonstrated a better QoL due to improvement of the depression-related symptoms (15). In conclusion, most of the available data indicate that CTO-PCI carries advantages in terms of symptom improvement compared with drug therapy alone. In patients without symptoms, ischemic burden evaluation is recommended, and CTO recanalization is indicated if ischemia is present in ≥10% of the left ventricle (LV) mass (8, 16).

One of the most investigated aspects of CTO-PCI is the possible improvement of LV systolic function. The REVASC trial showed no differences at 1 year between CTO-PCI and medical therapy in terms of changes in segmental wall thickening (SWT) in the CTO territory (17). However, several factors could have influenced these results. First, the revascularization of non-CTO lesions in the group treated with medical therapy may have increased the collateral flow, leading to recovery of areas of dysfunctional myocardium subtended by the occluded coronary artery in the group of patients without CTO-PCI. This speculation derives from a subgroup analysis showing that improvement in SWT occurred only in patients undergoing CTO revascularization who had less complex CAD (SYNTAX score <13). Second, another trial limitation could have been the lack of myocardial viability assessment with cardiac magnetic resonance (CMR) imaging. Indeed, a previous study demonstrated that SWT improved significantly only in patients with <75% transmural myocardial infarction (MI) at CMR indicating a significant remaining viable myocardium (18). Third, the mean LV ejection fraction (LVEF) of patients included in the REVASC study was normal (17). In this regard, a recent meta-analysis of 34 studies with 2,804 patients showed that a successful CTO-PCI is associated with a significant improvement of LVEF, especially in patients with lower baseline values (19).

A higher risk of ventricular arrhythmia or appropriate ICD shock has been reported in patients with CTO of an infarct-related coronary artery (IRA) (20). Ventricular arrhythmias in patients with a previous MI arise in the myocardial area surrounding the fibrous scar (21). In patients with CTO, hypoperfusion in this area could represent an arrhythmic substrate that may favor the occurrence of life-threatening arrhythmias. Indeed, a recent meta-analysis that assessed ventricular arrhythmias in patients with CTO has shown that an occluded vessel is associated with an increased risk of ventricular arrhythmia and all-cause mortality (22). Therefore, revascularization of IRA-CTO may generate positive electrical remodeling and reduce the arrhythmic risk by restoring blood flow in the viable myocardium close to the fibrotic scar.

There is a great discordance in the literature regarding hard end points such as mortality and major adverse cardiac events (MACE) between observational studies and randomized clinical trials. In the DECISION CTO trial, which assessed all-cause mortality, MI, revascularization, stroke and MACE, no advantages were found between the PCI group and patients treated only with drugs (11). However, a further limitation of the study, in addition to those already mentioned, was the exclusion of patients with a LVEF <30% who are those likely benefitting more from revascularization (23). In contrast, data from several registries showed an increase in survival in patients undergoing successful CTO recanalization compared with those with unsuccessful PCI (24, 25). A meta-analysis of 25 studies including 28,486 patients (26) showed a lower incidence of death, stroke, coronary artery bypass grafting and recurrent angina associated with CTO-PCI as compared to failed procedures. Similarly, in a more recent meta-analysis Li et al. reported possible benefits in terms of all-cause mortality, cardiac death and MACE in patients undergoing CTO revascularization in comparison with those treated with medical therapy (27). Similarly, although not powered for clinical end points, the REVASC study showed that at 12 months the CTO- PCI group had a lower risk of MACE, defined as total mortality, MI and any clinically driven repeat revascularization compared to the group with medical therapy alone (17). In addition, a recent study suggested that patients with no residual ischemia and extensive ischemic burden reduction after CTO-PCI had lower risk of all-cause death and non-fatal MI with a follow up of 2.8 years (28). Finally, Taek Kyu Park et al. (29) observed that CTO- PCI might reduce a 10-year rate of cardiac and all-cause death compared with optimal medical therapy, as well as that of acute MI and any revascularization.

## Imaging Modalities

### Echocardiography

#### Echocardiography for Pre-procedural Assessment

In patients with coronary artery disease (CAD) and in particular in those with CTOs, echocardiography provides important information on global LV function and regional wall motion abnormalities at rest. In order to make a correct indication for CTO revascularization, it is essential to differentiate transmural infarcted myocardium, which cannot benefit from reperfusion, from areas of hibernating but viable myocardium. In this context, transthoracic echocardiography is usually the first technique for myocardial viability assessment. Rosner et al. (30) demonstrated that evidence of normokinetic or slightly hypokinetic myocardium by means of wall motion scores and longitudinal strain measurement has a good negative predictive value for excluding transmural scar, even without the use of dobutamine stress echocardiography. Accordingly, in the latest consensus document for the management of CTO (3) it was agreed that the presence of normal myocardial function or hypokinesia in the CTO territory should be interpreted as a sign of myocardial viability and therefore, when symptoms are present despite optimal medical therapy, the revascularization procedure is indicated. Of note, the Rosner et al. study also showed that severe regional myocardial dysfunction by stress echocardiography is not always a manifestation of a transmural scar, suggesting that akinesia in a myocardial area subtended by a CTO should be further evaluated with other imaging techniques to detect viability and to guide therapeutic decisions. Dobutamine echocardiography is an accurate and reproducible method for detecting hibernating myocardium that may predict functional recovery after PCI (31). Myocardial segments hibernating at rest can improve contractility, showing the so-called “contractile reserve,” with low doses of intravenous dobutamine (5–10 micrograms/kg/min), while high doses of dobutamine (up to 40 micrograms/kg/min) can cause LV function worsening due to reduced coronary flow reserve. This biphasic response to dobutamine infusion is frequent in case of hibernating myocardium and seems to predict LV function improvement with high predictive value, as shown by Afridi et al. (32). In a systematic review of 37 studies that looked at different techniques that can predict regional and global improvement of function after revascularization in chronic CAD, Bax et al. reported that low dose dobutamine echocardiography showed the highest predictive accuracy (33). Transesophageal and transthoracic echocardiography may also play a role in the direct identification of CTOs. High sensitivity and specificity of transesophageal echocardiography were demonstrated in the detection of stenotic and occlusive coronary lesions using a modified continuity equation (34). In a study of 110 patients, occlusions of the left anterior descending (LAD) coronary artery and the right coronary artery (RCA), but not of the circumflex (CFX) coronary artery were demonstrated by transthoracic echocardiography with high sensitivity and specificity using retrograde flow in the epicardial and intramyocardial collaterals (35). One of the factors that may predict the success of CTO-PCI is the presence of well-developed collaterals, even if this condition does not seem to guarantee an advantage on survival and prognosis (36). Pizzuto et al. (37) measured in 51 patients the collateral flow reserve in occluded arteries with transthoracic coronary Doppler echocardiography. The measurement of collateral flow reserve (the ratio between hyperemic, during venous adenosine infusion, and baseline diastolic velocity of the stenotic vessel) was feasible and correlated directly with the angiographic evaluation of collaterals and the number of diseased vessels and was found to be inversely related to stenosis of the non-occluded arteries that provide the collaterals. Myocardial contrast echocardiography (MCE) is also a potentially useful method for estimating the microcirculation. Myocardial blood flow measured by MCE and in particular the plateau acoustic intensity, which represents the volume of myocardial flow, and the wall motion score index appear to correlate well with the flow of collateral vessels assessed by coronary angiography (38).

#### Echocardiography for the Detection of Procedural Complications

Transthoracic echocardiography provides a valid support for a rapid and easy identification of possible complications during and immediately after the revascularization procedure. Coronary perforation represents one of the most feared complications of CTO-PCI with an estimated incidence of 3% (39, 40). It can be responsible for pericardial effusion and tamponade, requiring emergency pericardiocentesis, and in some cases cardiac surgery. Echocardiography plays a key role in the evaluation of pericardial effusion and provides a prompt diagnosis of a life-threatening tamponade. Another well-known complication of CTO-PCI is perforation of a collateral vessel that can occur in 3–7% of cases (39, 40). Some perforations can progress to become septal hematomas, which in turn, can cause hemodynamic compromise through left-sided or biventricular outflow obstruction that can be promptly identified by transthoracic echocardiogram providing operators with detailed information needed for the correct management of the complication (41).

#### Echocardiography for Post-procedural Evaluation of LV Function

A successful percutaneous treatment of a CTO can improve LV systolic function (42). In a study of 43 patients with CTO treated with PCI, global longitudinal strain assessed with two- dimensional speckle tracking echocardiography (2D-STE) improved from the first day after the procedure, while LVEF tended to improve 3–6 months after the procedure (43). Meng et al. in a recent study of 63 patients with a single CTO demonstrated that at 2-year follow-up only global longitudinal strain and LV systolic function assessed with 2D-STE showed a statistically significant improvement in the group treated with PCI compared to the group receiving medical therapy only (44).

#### Future Perspective

The introduction of three-dimensional speckle tracking echocardiography (3D STE) may overcome some of 2D STE limitations, such as the need to acquire multiple images and out-of-plane speckle motion (45) improving reproducibility and accuracy. Furthermore, software improvements allowing tissue characterization and identification of myocardial scars may increase the use of transthoracic echocardiography for diagnostic and prognostic evaluation of patients with CTOs (46).

### Coronary Computed Tomography Angiography

Coronary Computed Tomography Angiography (CCTA) is emerging as an essential tool in the management of CTOs, from pre-procedural assessment, intraprocedural guidance to follow-up. When compared to other imaging modalities, it is the only non-invasive tool playing a crucial role in the practical guidance of revascularization procedures. Indeed, it is considered as the most comprehensive non-invasive imaging modality for CTO pre-procedural assessment and treatment planning that can dramatically increase the likelihood of successful PCI, especially in case of complex coronary lesions and previous unsuccessful attempts of revascularization.

#### CCTA for Pre-procedural Assessment

In the pre-procedural assessment of CTOs, CCTA is a potential “one stop shop” to assess anatomy, perfusion and viability in one single examination (47). It allows visualization and evaluation of the entire coronary tree including the CTO lesion, which appears as a “missing segment” when using dual injection at invasive coronary angiography. This is particularly useful in long and tortuous CTOs and in ostial occlusions, in which the definition of the course and the features of the occluded segment are key information for PCI success (48). A precise mapping of lesion tortuosity and distal vessel anatomy may be assessed using 2- and 3-dimensional reconstructions from any arbitrary angle, allowing an accurate measurement of the length, cross-sectional area and diameter of the CTO. Moreover, CCTA provides anatomical details of the atherosclerotic plaque, being highly accurate in defining the presence, location and extent of calcifications and in the description of the morphology of the proximal and distal CTO caps. These anatomical features strongly influence treatment strategy and materials selection for the interventional procedure. In case of heavily calcified lesions, the need for additional lesion pre-treatment such as rotational atherectomy or intravascular lithotripsy can be anticipated. Therefore, the anatomic information provided by CCTA before getting into the catheterization laboratory allow adequate procedural planning, potentially reducing procedural time and contrast dose, which are the most frequent reasons for stopping a CTO-PCI attempt.

A pitfalls of CCTA is the limited spatial resolution that may lead to inaccuracy in differentiating a CTO from an artery subocclusion and inability to visualize collateral vessels, which can be seen only with high-quality exams if the collateral diameter is >1.0 mm. The identification of a CTO could be increased by integrating multiple parameters, including ≥9-mm lesion length (49), presence of an adjacent side branch (most CTOs are imaged as “inter-bifurcation disease”), bridging collaterals and a blunt stump. Intracoronary attenuation-based analyses of CCTA may provide non-invasive functional and anatomical assessment of coronary collateral flow and may detect flow direction, predicting angiographically well-developed collateral vessels, refining the evaluation of complex coronary circulation in patients with CTO (50).

In addition to coronary anatomy evaluation, CCTA could also perform functional assessment of myocardial perfusion and viability by application of appropriate scanner protocols. However, routine use of CCTA for this purpose is not currently available.

#### CCTA and Prognosis

The detection of CTO at CCTA, which occurs in 6.2% of CAD patients undergoing this non-invasive imaging exam, is associated with a 2-year mortality similar to moderate-to-severe CAD (51). The success rate of CTO-PCI was until recent times about 75–80% (48); nowadays, the marked technological advances in terms of devices, techniques and operator experience have increased the procedural success over 90% but obviously there remains a gap between non-CTO and CTO procedures. Unsuccessful attempts of revascularization are associated with worse outcome (30-day MACE: 14.8 vs. 5.5%), mainly due to a significantly higher rate of MACE in the immediate period following the procedure (52). Therefore, careful selection of patients who are most likely to benefit from revascularization and have a good chance of a successful PCI is essential. CCTA offers the unique opportunity to non-invasively assess anatomical features of CTO that have been shown to predict PCI failure. Calcifications, a well-known hallmark of complex CTO, can be easily detected, localized and quantified by CCTA. Cross-sectional calcium area ≥50% of the vessel area, rather than calcium length, is a strong predictor of lesion crossing difficulty (53). The best cut-off value proposed is ≥54% (54). However, calcium length may influence the subsequent technical steps that follow each other during a contemporary recanalization procedure. The location of calcifications, whether at the proximal or distal cap, could influence the choice of the technical approach and material used. However, a study by Ehara et al. (55) demonstrated that the most prominent independent predictor of guidewire crossing failure was bending (defined as an angle >45° either at the occlusion site or proximal to the occluded segment), which can be missed on coronary angiography and is easily identified on CCTA. With respect to the predictive value of anatomical features, bending is followed by vessel shrinkage, i.e., an abrupt narrowing (<1-mm cross-sectional diameter) of the occluded segment that is indicative of negative remodeling and predictive of failure using an anterograde approach (55). In addition, a blunt stump morphology and the presence of multiple occlusions hampers successful guidewire crossing and passage into the distal true lumen.

Two computed tomography (CT)-based scoring systems, CT-RECTOR and KCCT, combine the previously described anatomical features with clinical characteristics to estimate the complexity of CTO-PCI and to predict the probability of successful guidewire crossing within 30 min. Both scores showed better predictive value than the J-CTO angiographic score (56, 57).

#### CCTA in PCI Guidance and Future Perspective

The new frontier is the application of CCTA directly in the catheterization laboratory for real time PCI guidance. Advancements in software for identification of centerline and vessel contour allow the fusion of 3D reconstructed CCTA images onto live fluoroscopy images during coronary angiography. To compensate for breathing and heart beating, the extracted CCTA images of the coronary vessels are matched to the diastolic images of the invasive series using bifurcation points as markers. The co-registration helps the identification of projections that minimize foreshortening of the coronary segment of interest and vessel overlap. Moreover, it allows the visualization of the CTO morphological features and the geometry of the luminal path to indicate guidewire direction and advancement. This approach resulted is a significantly higher success rate of CTO-PCI compared with procedures performed without CCTA (58). A more technologically complex approach with continuous co-registration is now possible thanks to dynamic 4D roadmap acquired from multiple phases during the cardiac cycle. Integration of 4D multi-slice data, however, is only achievable off-line for a single respiratory phase. Respiratory gating requires the integration of dedicated sensors using small magnetic fields, such as those employed in the MPS-system from Mediguide (Haifa, Israel) (59). Another innovative technology to guide CTO-PCI is based on precise stereotactic localization and control of the guidewire tip using magnetically enabled guidewire while crossing the lesion. The magnetic navigation technology developed by Stereotaxis, the Niobe® Magnetic Navigation System (MNS, Stereotaxis, Inc., St. Louis, MO, USA) is based on the creation of a uniform magnetic field of 0.08 Tesla within the patient chest by two permanent external magnets placed on either side of the fluoroscopy table. In this magnetic field, the tip of the guidewire, provided with a tiny magnet, can be precisely directed with a full 360° omni-rotation. A virtual roadmap of the coronary tree acquired from 3D volume-rendered CCTA images displays the changes of the magnetic vector as the guidewire is advanced and indicate its position. This technology, in limited experiences, has been shown to increase safety and effectiveness of CTO-PCI, along with a reduction in contrast dose (60). Recently, a novel technology uses a more complex CT approach, which relies on the integration of flat panel detectors positioned on the C-arm of the X-ray machine. Therefore, image acquisition and reconstruction are possible directly during PCI, without the need of patient transfer.

The main limitation of the intra-procedural application of CCTA consists in the need of additional radiation dose and contrast use in patients undergoing PCI. However, the latest CT-scanners have dramatically reduced the amount of radiation and contrast and, at the same time, improved image quality. Since CCTA-guided PCI resulted in reductions of procedural time and complication rates, it could be hypothesized that the net sum of radiation and contrast during these procedures is equal or even lower than that of patients not undergoing CCTA. Future studies will be needed to prove this hypothesis. Moreover, stress myocardial CT perfusion (CT-MPI) and fractional flow reserve derived from CCTA (FFRCT) have been introduced in clinical practice as new tools for evaluating the functional relevance of coronary stenoses, with the possibility to overcome the main CCTA drawback, i.e., anatomical assessment only (61). Conventional CT scanners can be applied for stress myocardial perfusion imaging to diagnose inducible myocardial ischemia, although better image quality can be achieved with more contemporary technology. As for the other stress myocardial imaging modalities, during pharmacologically induced myocardial hyperemia (adenosine, regadenoson, dipyridamol), obstructive CAD causes relative hypoperfusion, which can be visualized through injection of contrast agent. In the specific field of CTO, CT-MPI seems particularly useful, by combining anatomical information on lesion characteristics from CCTA with the presence and extension of ischemia from perfusion assessment and demonstrated high and similar diagnostic performance vs. other non-invasive stress tests to identify flow-limiting coronary lesions at invasive FFR (62–64). Finally, some group of researchers are developing a dedicated tool for a full, real-time integration of anatomical (calcium and atherosclerosis) and functional (FFRCT) CCTA-derived information into the cath lab for the guidance of PCI (65).

### Cardiac Magnetic Resonance

Cardiac Magnetic Resonance (CMR) is a high-resolution non-invasive imaging technique that can assess regional and global LV function, as well as detect the presence and the extent of MI and ischemic burden before PCI. CMR could also provide a careful post-procedural evaluation to assess CTO-PCI efficacy.

#### CMR for Pre-procedural Assessment

CMR is the non-invasive gold standard for the measurement of LV volume and LVEF, showing very high reproducibility, and low intra- and inter-observer variability (66). CMR is also an excellent tool for the evaluation of myocardial viability by means of late gadolinium enhancement (LGE) extent and the response to inotropic drug such as low-dose dobutamine. Moreover, CMR can easily assess ischemia by evaluating perfusion defects and wall motion abnormalities, which are important to define the indication for a revascularization procedure in the presence of a CTO (67).

According to the CARISMA-CTO study (67), viability can be defined as <50% of LGE in the region of interest or as an improvement in segment function >1 grade during low-dose dobutamine infusion. LGE has become the most widely used technique for tissue characterization and is considered the cornerstone of myocardial viability assessment. The gadolinium-based contrast agents used for LGE may differentiate viable myocardium from scar in different clinical settings, including MI. The degree of LGE transmurality is related to the time of myocardial ischemia and to the potential functional recovery following revascularization (68). Generally, a transmural extent of 50% is considered a cut-off value to predict contractile function recovery in patients who undergo coronary revascularization. Nakachi et al. showed that, in the CTO territory, longitudinal circumferential strain significantly improved in segments with a LGE extent <50% after CTO-PCI, but not in segments with a transmural extent of LGE >50% (69). However, poor data are available regarding the optimal cut-off value of the transmural extent of LGE at CMR to detect myocardial segments that will functionally recover after CTO recanalization. Although LGE has high sensitivity to detect scar tissue, a study including 71 patients with 122 CTO showed that about one third of segments showing a transmural scar had inside them hibernating myocardium detected by ^99m^Tc-sestamibi and ^18^F-FDG imaging. Therefore, CMR seems to be less accurate than CT scan in terms of hibernating myocardium detection (70).

Several CMR parameters have been evaluated to predict LV functional recovery after CTO-PCI. Among them, extracellular volume (ECV) fraction proved to be superior to both LGE and rim thickness for predicting the improvement of regional and global LV function improvement 6 months after revascularization in patients with CTO (71). In addition, novel T1 relaxation time maps (“T1 mapping”) offer a quantitative evaluation of diffuse myocardial fibrosis, hence overcoming the limitation of traditional LGE sequences when myocardial abnormalities are present (72).

Regarding the diagnosis of reversible ischemia, stress CMR represents an excellent alternative to other non-invasive stress tests to detect perfusion defects and regional wall motion abnormalities (WMA). Adenosine and high-dose dobutamine stress CMR allows to identify perfusion anomalies and to quantify ischemic burden in CAD patients, especially in those with CTO. Stress CMR is accurate in detecting inducible ischemia due to flow-limiting stenosis of the epicardial coronary arteries (73–75), showing perfusion defects and WMA, and in characterizing hibernating myocardium. Dobutamine stress CMR may also predict recovery of function after revascularization in patients with chronic regional WMA. Functional improvement of hypokinetic or akinetic segments during low-dose dobutamine (5–20 μg/kg/min) has been shown to be more specific than LGE assessment, especially when LGE transmurality is intermediate (<75%) (76). Some groups use a low- and high-dose dobutamine protocol to assess the presence of a biphasic response that may indicate hibernating myocardium. However, most clinicians would use either type of response as a sign of viability to maximize the test sensitivity.

#### CMR and Prognosis

Several CMR studies have evaluated patients undergoing CTO-PCI, in order to identify which patients may benefit the most from the procedure. In patients undergoing PCI with evidence of ischemia and viability on MRI, an improvement in LVEF and volume have been observed. In patients with CTO, Baks et al. (77) and Kirschbaum et al. (18) demonstrated that after PCI there were both early and late improvements in regional LV function in the perfusion territory of CTO that were related to the transmural extent of MI (LGE extent) on pretreatment CMR imaging. Bellanger et al. (78) showed a significant correlation between the number of viable segments within the infarct zone and end-systolic volume and LVEF improvement at follow-up. Fiocchi et al. (79) showed that segmental contractility improvement during low-dose dobutamine might predict LV function recovery at 6 months after PCI in a small cohort of patients. Bucciarelli-Ducci et al. (80) in 32 patients selected for CTO-PCI based on myocardial viability (LGE <75%) and myocardial ischemia, demonstrated that revascularization significantly reduced inducible ischemia, favored reverse remodeling and improved quality of life. Cardona et al. (81) showed that one third of their patients with successful CTO-PCI also underwent PCI of non-occlusive coronary stenoses. No difference in the degree of LVEF improvement was seen when this group was compared with patients who did not undergo non-CTO-PCI, suggesting that changes in LV function parameters after successful CTO-PCI were derived from CTO recanalization. Rossello et al. (82) proposed a novel CMR ischemic burden index based on the characteristics of perfusion defects. High scores were associated with a greater improvement in exercise tolerance. In contrast, the REVASC trial showed that after successful CTO-PCI there were no improvements in CMR parameters even though there was a reduction in clinical end points compared to medical therapy (17). The CARISMA study (67) proposed a multi-parameter CMR protocol tailored on patients suitable for CTO-PCI to evaluate viability with LGE and ischemia with a perfusion and stress study with low- and high-dose dobutamine for identifying those who could most benefit from CTO-PCI.

#### Future Prospective

The introduction of 3D CMR perfusion imaging represents a promising tool for measurement of myocardial blood flow and an alternative technique to single photon emission computed tomography (SPECT) or positron emission tomography (PET). Compared to the conventional 2D multislice perfusion imaging, 3D CMR allows quantification of the percentage of ischemic myocardium and reduces the scan time by a simultaneous acquisition of all slices at the same point of the cardiac cycle (83). The strain technique was recently applied to CMR in the field of myocardial deformation assessment, facilitating the accurate identification of patients at high risk of future cardiac events who may be candidate of revascularization procedures (84).

### Nuclear Cardiac Imaging

Nuclear cardiac imaging can be useful for CTO pre-procedural assessment in terms of evaluation of myocardial viability and ischemia by myocardial perfusion imaging (MPI) and scintigraphy (MPS) studies during stress or rest. MPS can be obtained by two main techniques: single photon emission computed tomography (SPECT) and positron emission tomography (PET). During the tests, a radioactive isotope tracer administered intravenously reaches the viable myocardial cells. Subsequently, photons or positrons are emitted from the myocardium in proportion to the extent of tracer uptake, which correlates with perfusion. Several studies demonstrated that myocardial perfusion defects, found in SPECT MPI, might predict MACE (85). At present, during a SPECT acquisition it is preferred to use a technetium-based tracer instead of the older thallium-201-chloride. The use of technetium, coupled with high count rates gated SPECT allows LV function and myocardial perfusion to be assessed with superior image quality and lower radiation dose (86).

For CTO evaluation, Wright et al. demonstrated that evidence of ischemia on MPI by SPECT with Technetium (^99m^Tc) sestamibi accurately predicted MACE (death, MI, unstable angina), whereas the demonstration of distal collateralization at angiography alone failed to predict freedom from ischemia and MACE (87).

Moreover, SPECT is a very effective imaging technique for distinguishing viable from non-viable myocardium and for predicting contractile function recovery following revascularization with a mean sensitivity of 84% and mean specificity of 77% (88). This led to a better selection of patients who may derive the higher benefit in terms of both LV function and prognosis.

In PET studies, N-13 ammonia and rubidium-82 are tracers used for rest MPI evaluation, whereas 15O-labeled water and 18F-labeled are agents used to test cardiac glucose metabolism. Preserved glucose metabolism is a sign of cardiac viability in regions without normal resting perfusion. The absence of 18F-FDG uptake, on the other hand, implies a non-viable myocardium.

Compared with SPECT, PET MPI carries various technological advantages, including better space resolution, accurate attenuation correction, a technique that removes soft tissue artifacts, and lower radiation exposure. These benefits are especially important in viability studies because they help identify the existence, amount, and severity of myocardial scars. Moreover, PET MPI is considered the gold standard for non-invasive MPI since it can obtain a quantitative analysis of myocardial blood flow (MBF) and coronary flow reserve using a pharmacological stress (89). These findings allow assessing the condition of both the epicardial and microvascular circulation, providing an absolute quantification of myocardial perfusion.

Using [^15^O]H_2_O PET performed prior and after successful PCI of CTO or non-CTO lesions in patients with preserved LVEF, Schumacher et al. demonstrated that, although myocardial perfusion findings were slightly more hampered in CTO patients before and after PCI, CTO-PCI improved absolute myocardial perfusion and reduced the extent of the perfusion defect similarly to PCI of hemodynamically significant non-CTO lesions, emphasizing that this a useful diagnostic tool in CTO patient selection (90).

#### Future Perspective

The introduction of hybrid PET/CCTA allows for the precise identification and assessment of myocardial ischemia and viability in conjunction with the evaluation of coronary morphology. As a result, hybrid imaging will be helpful in the clinical work-up of CTO patients to better identify eligibility and plan the strategy of revascularization.

## Discussion and Conclusion

**Figure 1:**
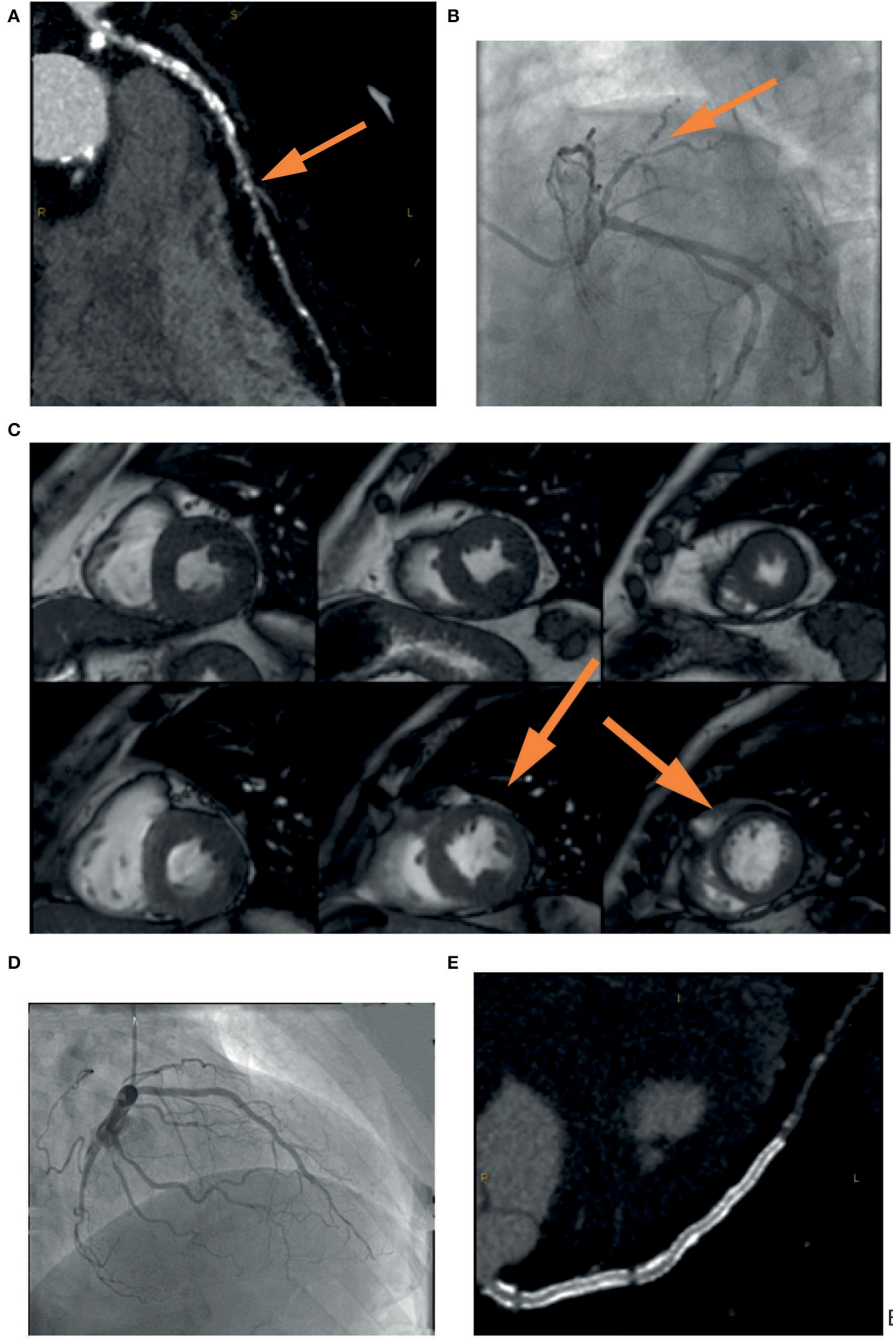
Example of the usefulness of multi-modality imaging in placing a correct indication for CTO percutaneous treatment. A 72-year-old patient performed a coronary computed tomography angiography (CCTA) **(A)** that showed severe three-vessel disease with occlusion (*yellow arrow*) of the left anterior descending (LAD) coronary artery. Coronary angiography **(B)** confirmed the presence of a calcified lesion occluding the LAD (*yellow arrow*) that was filled by means of collateral circulation. In consideration of the lack of symptoms and the unfavorable anatomy, it was decided not to proceed with a procedure without first assessing ischemia in the territory of the vessel. Cardiac stress magnetic resonance **(C)** showed inducible ischemia and myocardial viability in the mid-apical segment of the anterior wall and the interventricular septum (*yellow arrow*). Coronary angioplasty was then performed with implantation of a drug-eluting stent in the left main and LAD with an excellent result **(D)**. Vessel patency without restenosis was confirmed by coronary CCTA at follow-up **(E)**.

**Figure 2:**
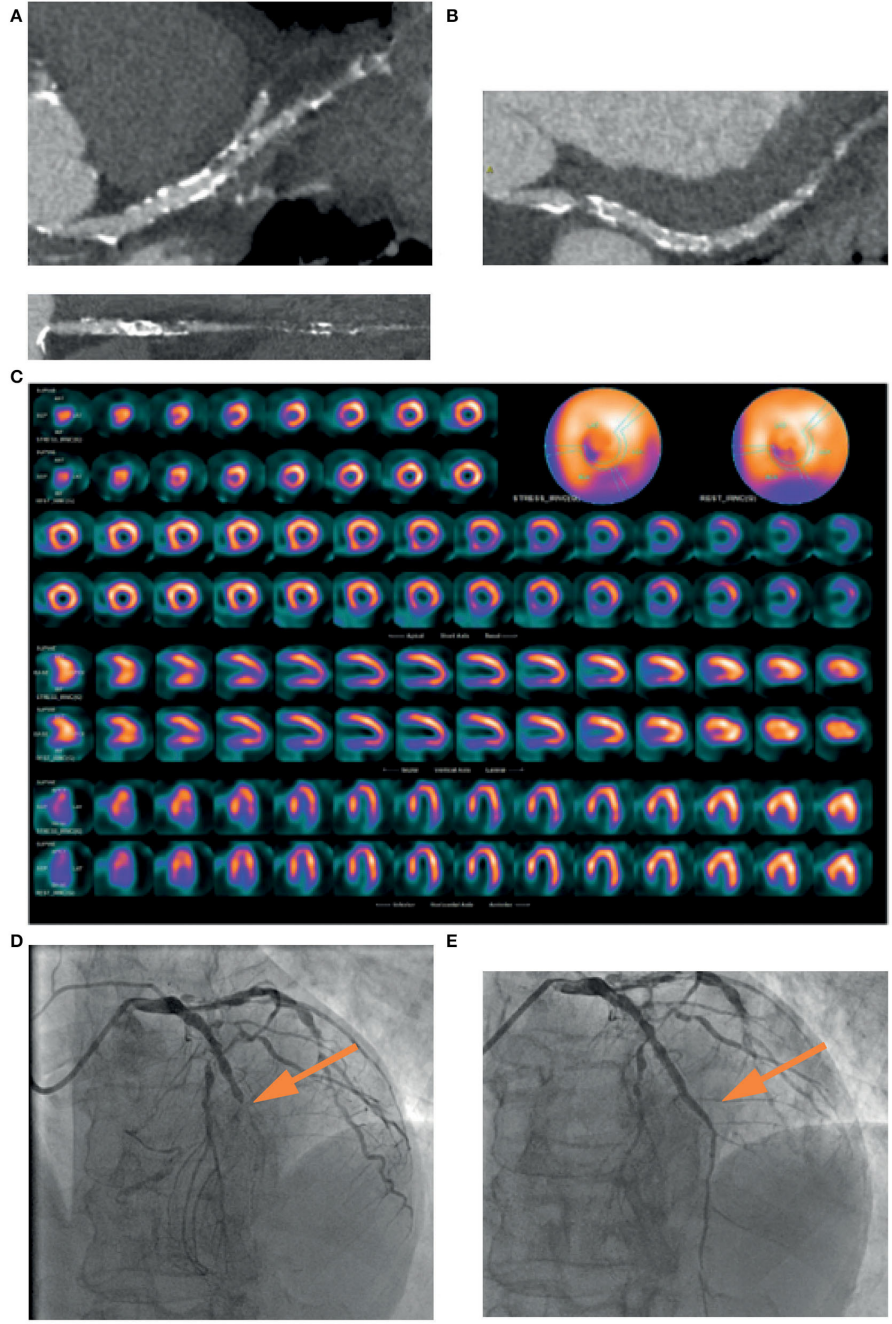
A 74-years-old patient, symptomatic for chest pain, performed a coronary CT, which showed a mild stenosis of left main and a diffuse severe stenosis of mid-distal left anterior descending (LAD) coronary artery **(A)**. Circumflex artery had a significant ostial stenosis, with sub-occlusion of mid-distal portion **(B)** and right artery was occluded at the mid portion **(C)**. SPECT showed perfusion defect at the apical septum and at infero-lateral mid-basal wall **(D)**. Coronary angiography confirmed the presence of sub-occlusion (*yellow arrow*) of the middle section of the LAD coronary artery **(E)**, treated with coronary angioplasty and placement of a medicated stent (*yellow arrow*), with a good final angiographic result **(F)**.

At the present time, there are no clear indications from the literature on the preferred imaging method or on the steps to be followed in the pre-procedural evaluation of patients with CTOs (Figures 1, 2). This suggests that knowledge of the limits and advantages of each method represents the starting point for a correct approach and appropriate management.

Stress echocardiogram is an easily accessible and low-cost technique that does not require the administration of contrast or radiation. On the other hand, it is operator-dependent and requires good image quality, even though it is likely that in the near future its accuracy could increase also favored by regional strain evaluation.

Cardiac CT, which requires the administration of radiation and contrast medium, is particularly indicated in the pre-procedural study of complex coronary anatomy. Of note, the latest scan generation has reduced the estimated radiation dose to 2–4 mSv. Stress myocardial computed tomography perfusion (CTP) allows an assessment of the functional relevance of coronary stenoses and could therefore provide information on myocardial viability. However, at present there are no specific studies of its application in patients with CTO.

Cardiac MRI does not use ionizing radiation and has good spatial resolution, sensitivity and specificity in the evaluation of myocardial viability. However, it has significant costs and may have lower sensitivity for hibernating myocardium identification.

SPECT imaging allows measuring LV function and simultaneously evaluating myocardial ischemia but it does not clearly differentiate between non-viable and hibernating myocardium. On the other hand, PET, thanks to the measurement of absolute myocardial flow and coronary flow reserve, has excellent diagnostic accuracy and high sensitivity in the evaluation of myocardial viability, with the limitation of 2–5 mSv radiation exposure and high cost.

**Figure 3:**
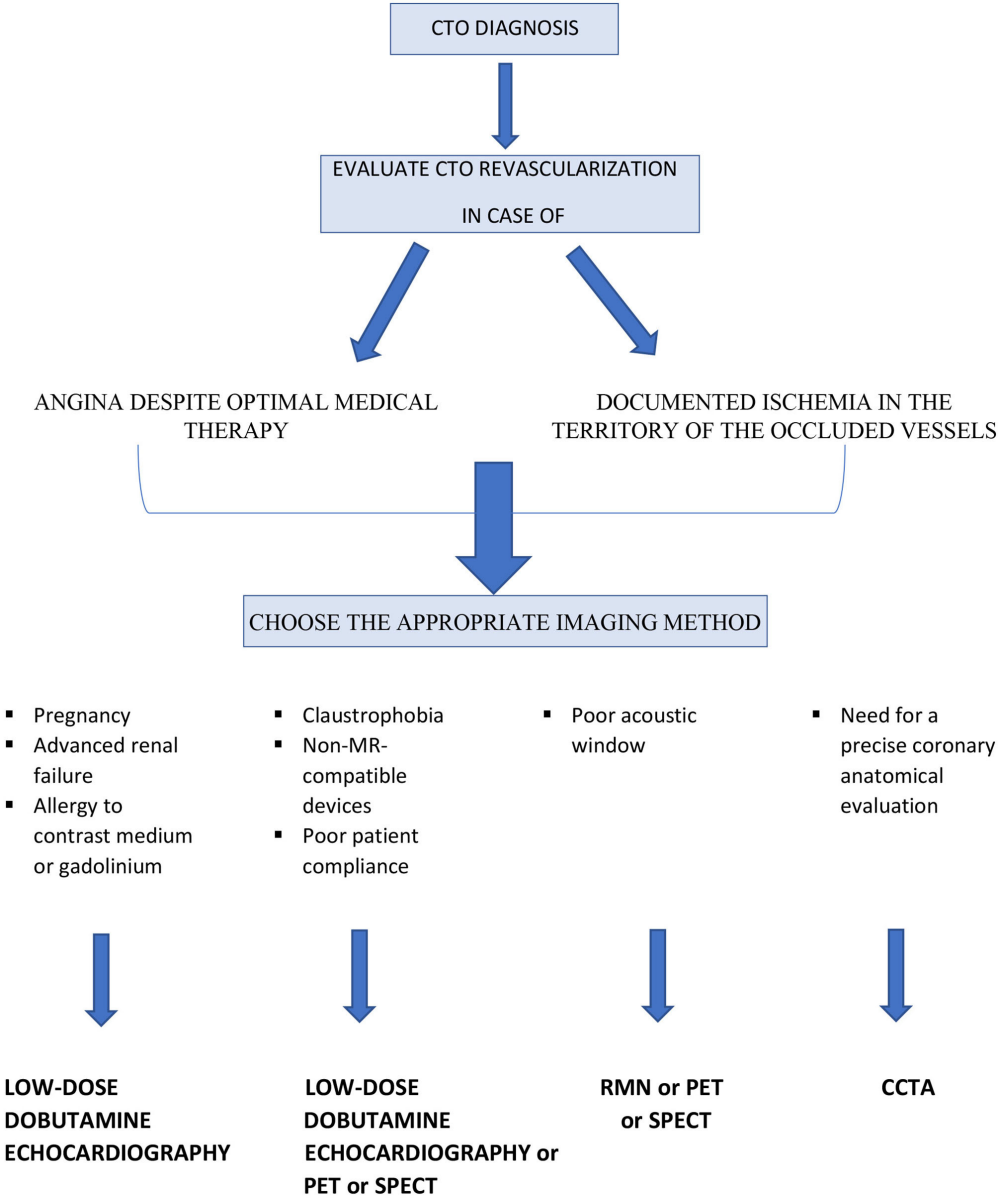
Flow chart to guide the choice of the appropriate imaging method in patients with CTOs.

Table 1 summarizes the main characteristics of each imaging method. The authors also propose a simple flow chart to guide the clinicians and the interventional cardiologist for selecting the more appropriate imaging modality in patients with CTOs (Figure 3).

**Table 1:** Main features of each imaging modality.

	**Low-dose dobutamine echocardiography**	**CCTA**	**CMR**	**PET**	**SPECT**
Radiation exposure (mSv) (91)	0	2–4 mSv	0	^18^FDG: 5-7 mSv ^13^NH_3_: 4 mSv	^99^TC: 20 mSv
Cost	+	++	+++	+++	++
Operator dependency	+++	+	+	+	+
Ischemia quantification (92)	YES	NO	YES	YES	YES
Viability sensitivity (95%CI) (91–93)	81% (80-82)	-	95% (93-97)	93% (91-95)	81% (78-84)
Viability specificity (95%CI) (91)	80% (79-81)	-	51% (40-62)	58% (54-62)	66% (63-69)

In conclusion, coronary CTOs represent a diagnostic and therapeutic challenge for the cardiologist. Multimodality imaging and a multidisciplinary team approach are essential for an individualized decision-making and effective treatment planning.

## Author Contributions

All authors listed have made a substantial, direct, and intellectual contribution to the work and approved it for publication.

## Conflict of Interest

The authors declare that the research was conducted in the absence of any commercial or financial relationships that could be construed as a potential conflict of interest. The reviewer FC declared a shared affiliation, though no other collaboration, with one of the authors DN to the handling Editor.

## Publisher's Note

All claims expressed in this article are solely those of the authors and do not necessarily represent those of their affiliated organizations, or those of the publisher, the editors and the reviewers. Any product that may be evaluated in this article, or claim that may be made by its manufacturer, is not guaranteed or endorsed by the publisher.
